# ‘Predatory’ open access: a longitudinal study of article volumes and market characteristics

**DOI:** 10.1186/s12916-015-0469-2

**Published:** 2015-10-01

**Authors:** Cenyu Shen, Bo-Christer Björk

**Affiliations:** Information Systems Science, Hanken School of Economics, PO Box 479, Arkadiankatu 22, Helsinki, 00101 Finland

**Keywords:** Open access, Scientific publishing

## Abstract

**Background:**

A negative consequence of the rapid growth of scholarly open access publishing funded by article processing charges is the emergence of publishers and journals with highly questionable marketing and peer review practices. These so-called predatory publishers are causing unfounded negative publicity for open access publishing in general. Reports about this branch of e-business have so far mainly concentrated on exposing lacking peer review and scandals involving publishers and journals. There is a lack of comprehensive studies about several aspects of this phenomenon, including extent and regional distribution.

**Methods:**

After an initial scan of all predatory publishers and journals included in the so-called Beall’s list, a sample of 613 journals was constructed using a stratified sampling method from the total of over 11,000 journals identified. Information about the subject field, country of publisher, article processing charge and article volumes published between 2010 and 2014 were manually collected from the journal websites. For a subset of journals, individual articles were sampled in order to study the country affiliation of authors and the publication delays.

**Results:**

Over the studied period, predatory journals have rapidly increased their publication volumes from 53,000 in 2010 to an estimated 420,000 articles in 2014, published by around 8,000 active journals. Early on, publishers with more than 100 journals dominated the market, but since 2012 publishers in the 10–99 journal size category have captured the largest market share. The regional distribution of both the publisher’s country and authorship is highly skewed, in particular Asia and Africa contributed three quarters of authors. Authors paid an average article processing charge of 178 USD per article for articles typically published within 2 to 3 months of submission.

**Conclusions:**

Despite a total number of journals and publishing volumes comparable to respectable (indexed by the Directory of Open Access Journals) open access journals, the problem of predatory open access seems highly contained to just a few countries, where the academic evaluation practices strongly favor international publication, but without further quality checks.

## Background

### Introduction

The publishing of scholarly journals has, like so many other areas in business and society, undergone a radical transformation due to the emergence of the Internet. Mainstream publishers of subscription journals started publishing parallel electronic versions of their journals around the millennium shift [1] and today electronic delivery of big bundles of journals via e-licensing is the dominating business model.

A side-effect of this transformation was the prospect it offered for a more radical rethinking of revenue models. New innovative publishers repositioned themselves as service providers to the authors, publishing with them, rather than seeing themselves as content providers to readers. In this model, authors pay the publishers for their services, including that the articles become freely accessible to anybody with Internet access (open access, OA). Other than that, the peer review practices, layout, indexing, and so on, remain largely the same. A major difference is nevertheless that the journals are published only in electronic format and that the delay from submission to publishing is usually shorter compared to traditional scholarly journals.

Open access scholarly publishing also includes OA journals without publishing fees and subscription journals, which also make their electronic version freely available directly or after a delay [2]. In addition, the vast majority of subscription journals from leading publishers nowadays make individual articles available after payment, so-called hybrid OA [3]. Direct OA publishing is often called ‘gold’ OA. In addition, there is a ‘green’ route in which authors or third parties can legally make manuscript versions of articles published in traditional journals freely available on the Internet [4]. This can be done on the authors’ own webpages, or preferably in institutional or subject-based repositories.

The number of OA journals charging authors (using article processing charges, APCs) and the number of articles published by them has rapidly risen in the last decade, and some journals have reached a high scientific status in their field. Publishers have also started experimenting with novel forms of peer review, in particular in so-called ‘megajournals’, which only check for scientific rigor and validity, not for the significance of the results, which is left to the readers to decide [5]. The spectacular success of the leading megajournal, *PLOS ONE*, which publishes around 30,000 articles per year, shows that authors appreciate this model.

This study is, however, concerned with a peculiar sub-class of OA journals using APCs, made possible by the global reach and cost-effectiveness of the Internet. Publishers of this type of journal seem to be in the scholarly publishing business only in order to collect APCs and provide rapid publishing without proper peer review for authors who need publications in their CVs. The information on the Internet about the journals is often strongly misleading, and the publishers spam academics all over the globe with requests for submissions and reviews and for joining editorial boards.

Jeffrey Beall coined the phrase ‘predatory publishers’ to describe publishers of this sort of journal [6]. Another term that has been suggested is pseudo-journals [7]. Beall has also defined a long list of criteria for identifying such journals and produces a continuously updated index of publishers as well as individual journals fulfilling such criteria [8].

Predatory publishers have caused a lot of negative publicity for OA journals using APCs, partly due to the spam email that they constantly send out to researchers and partly due to a number of scandals involving intentionally faulty manuscripts that have passed their quality control. Predatory OA is regularly discussed and warnings are issued in academic journals, in particular in editorials of scholarly journals [9] and journals widely read by medical practitioners [10]. This indirectly makes it more difficult for serious OA journals to attract good manuscripts and get accepted to indexes such as Web of Science.

Since most of the reporting in the media about predatory OA has been concerned with individual cases and there have been very few scientific studies of the topic, the overall aim of this study was to: estimate the overall size of predatory publishing; examine how it has grown in the last few years; and measure key characteristics of this market.

### Earlier research

Reports of substandard or even nonsensical papers having been published in peer-reviewed journals have gained a lot of publicity through coverage in the popular press. In 2009, Phil Davis reported that he and a colleague had submitted a grammatically correct but nonsensical manuscript generated by a software program to Bentham’s *Open Information Science Journal*, and that he had subsequently received a mail stating that the article had been accepted for publishing, provided he would first pay the publication charge of 800 USD [11]. An experiment designed by the journalist John Bohannon, in which a spoof manuscript containing major methodological errors and other weaknesses was accepted by 157 journals and rejected only by 98, also caught the attention of the general media [12]. The problem with these types of studies is that they tell little about the scientific quality of the average papers in these journals. They do demonstrate that the peer review practices are often so deficient that just about any sort of paper could be accepted for publishing without revisions in many of these journals.

A few case studies of predatory journals have been reported. Djuric describes in detail the publishing pressures in Serbia, where the government requires publishing in journals having an ISI impact factor for academic appointments and even to obtain a PhD [13]. This has led to a niche market for some local publishers, which have managed to get their journals into Web of Science, in the wake of Thomson Reuter’s drive to index more regional journals during the latter half of the previous decade. Djuric sent a purposefully flawed manuscript to one such journal, in which several of his university colleagues had published recently, and got an acceptance the next day with instruction on how to pay the APC.

Lukić et al. discuss a number of cases of ‘hijacked’ journals. In such cases, the activities are directly fraudulent [14]. The hijackers create websites with the same names as respectable journals and then solicit manuscripts via spam email.

A particularly interesting but somewhat atypical case is offered by *Experimental & Clinical Cardiology* [15]. The journal had for 17 years been published by a respectable Canadian subscription publisher. The journal, which had a JCR impact factor (0.7), was purchased by investors of obscure background, changed the business model to OA, funded by an APC of 1,200 USD, and rapidly increased the number of articles from 63 in 2013 to over 1,000 in 2014.

The only published empirical qualitative study that we could find which sheds light on the dilemma of predatory publishing is the study by Omobowale et al. [16] who interviewed 30 academics from two Nigerian universities. A central finding was the difficulty of getting published in ‘Western’ journals, while at the same time, university administrations requiring ‘international’ publication; two factors that together have been strong drivers for the emergence of the market demand for ‘predatory’ publishing.

There have been a couple of published studies about the volume and other characteristics of predatory journals. Xia examined 297 journals listed in Beall’s list of standalone predatory journals, and found an average APC of 94 USD and a range of yearly articles of between 4 and 2,286 (mean 227 articles, median 86) [17].

Xia et al. also studied the origin of authors in seven pharmacological journals included in the above list and found a strong dominance of Indian authors, with Nigerian and Pakistani authors in second and third place [18]. Ezinwa Nwagwu and Ojemeni studied 34 journals published by Nigerian-based publishers, Academic Journals Inc. and International Research Journals, both focusing in biomedicine [19]. They found that 57 % of authors were from Asia and 28 % from Africa, with Nigeria, China and India being the leading countries.

### Research questions

The specific research questions of this study were:

- What is the current number of predatory journals (both active and empty)?
- What number of articles are published in them per year and how have these numbers evolved over the past few years?
- What is the distribution of articles over broad scientific fields?
- In what countries are they published?
- From what countries do the authors come?
- How much do they charge the authors for publishing?
- How rapidly do these journals publish?

## Methods

### Identifying predatory publishers

The first question to be asked is how to define a predatory publisher (as well as journal). For practical purposes it would have been impossible for us to construct a new or adapted list of criteria and then search the Internet for publishers and/or journals fulfilling these criteria. Instead, the work already done by Beall in compiling his index of predatory publishers as well as individual predatory journals was used as the starting point for empirical data collection. Beall has defined a detailed list of criteria [8] for determining if a publisher/journal is predatory. The list is rather long with 48 criteria, for either the publisher or individual journal, and is grouped under four major headings (editor and staff, business management, integrity and other). The criteria cover a vast array of direct and indirect indicators of the lack of a rigorous scientific quality control of the published articles as well as of the publishers trying to establish a reputable image in order to attract submissions. For instance, it is often very difficult to find out in which country the publisher operates in practice. At the same time, authors and institutions are often assumed to base their evaluation of journals at least in part based on the publisher’s location, with a preference for US and Western European locations. Another indicator is that some publishers have rapidly created vast portfolios of journals covering just about all fields of science, many of which lack content. A third is that many publishers advertise very rapid turnaround times from submission to publication, which would defeat the purpose of peer review by competent researchers.

Both of the lists (which are regularly revised) were downloaded on 1 September 2014 [20]. At that time, the list of publishers included 614 items and the list of individual journals had 416 items. The publishers of the latter were classified as single-journal publishers in our study, leading to a total number of 1,030 publishers as a starting point. The next step was to review each publisher’s website in order to count the number of journals published and to record the publisher’s country of origin. We excluded 64 publishers from the entire population for the reason that they had invalid links, published no journals or provided no journal-related information. Of the remaining 966 publishers, we found a total of 11,873 published journals. This preliminary analysis demonstrated the heterogeneity of predatory publishers in terms of their journal size; most publishers are relatively small with less than ten journals, but there are several publishers with large fleets of journals.

### Sampling

**Fig. 1:**
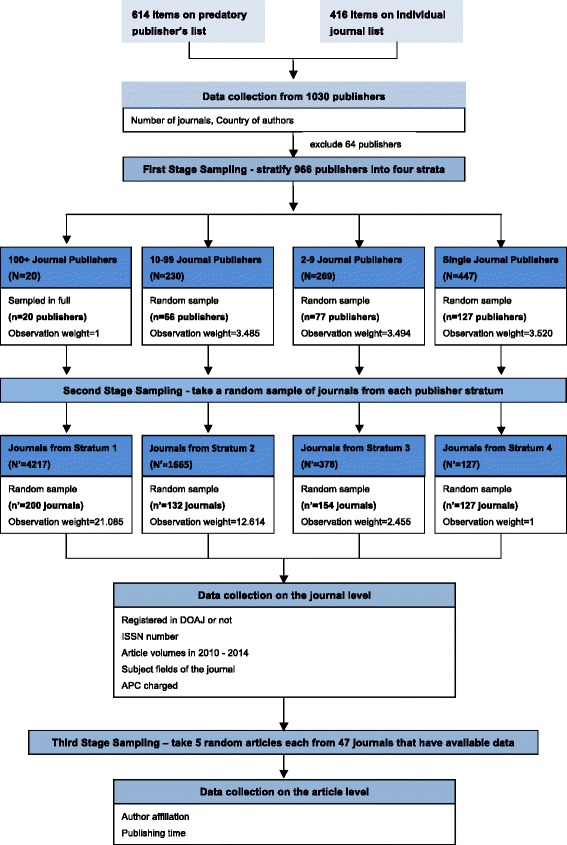
Sampling process

It would have taken a lot of effort to manually collect publication volumes and other data for all 11,873 journals, so the only practical solution was to make a sample of journals to generalize from. One option would have been a fully random sample, with each journal having the same chance of being selected. We suspected, however, that journals from small publishers often publish a much higher number of articles than those of large publishers, and this was verified in a small pilot test, using data from ten random journals from small and large publishers, respectively. Hence, a fully random sample would probably have resulted in an underestimation of the total number of articles, since journals from the large publishers with large journal portfolios would have dominated the picture and very few journals from single-journal publishers would have been included in the sample. Instead we chose a stratified multistage sampling based on the size of the publishers by first splitting the publishers into four size strata (100+ journals, 10–99 journals, 2–9 journals and single-journal) and then randomly sampling publishers within each of these strata. The sampling process is illustrated in Fig. 1.

In the first stage of sampling, we randomly selected a total of 290 publishers from the different strata. In the case of the 100+ stratum, we did not in fact sample but included all 20 publishers in that category. After that, a random number of journals were chosen among the included publishers. In the 100+ stratum, ten journals were sampled per publisher. Both in the 10–99 and 2–9 strata, we sampled two journals from each publisher. In the single-journal strata, considering that such journals are more likely to produce more articles, more journals (n = 127) than in the other strata were fully sampled so that more reliable results concerning the total article volumes from this stratum could be obtained. This resulted in a total sample of 613 journals.

Due to the use of multistage stratified sampling design, the sampling weight *W*_*ij*_ attached to each journal is equal to the reciprocal of its overall probability of selection, which is the product of the probability of selecting the *i*^*th*^ publisher at the first stage (*P*_*i*_) and the probability of selecting the *j*^*th*^ journal from the selected *i*^*th*^ publisher at the second stage $\left({P}_{j_{(i)}}\right)$. The sampling weight used in the analysis was calculated according to the following formula [21]:

$$ {W}_{ij}=\frac{1}{P_{i*{P}_{j_{(i)}}}} $$

### Data collection

The following data were extracted for each sampled journal: registered in the Directory of Open Access Journals (DOAJ) or not; ISSN number; subject field of the journal; article volumes in 2010–2014; and APC.

The results obtained from searching journals’ titles from Beall’s list, on the DOAJ website (doaj.org) were collected in order to estimate the proportion of current predatory journals included in the DOAJ. The discipline breakdown is based on a previous study [22]. In addition, we decided to introduce a new category called ‘general’ to represent the subject areas of journals that encompass more than one classified discipline. Finding out the APC was mostly straightforward, but some journals had very flexible charges depending on different factors, for instance, the number of authors, their countries (for example low-income, middle-income and high-income countries), identities (for example students, researchers, and so on), and the length and type of articles (for example review articles, research articles, and so on). To determine the average size of APCs charged by such journals, we studied ten articles from the journal, estimated the likely cost and then calculated the average using a method replicated from an earlier study [23]. All the APCs were counted based on the prices listed at the time of data collection. The currency used was the US Dollar (USD) and the prices given in currencies other than the USD were converted according to the exchange rate on Currency Converter [24].

We also wanted to estimate the average publishing speed (submission to publication) of predatory journals as well as the geographical spread of authors. For this purpose we collected five random articles for such journals where the submission and publication date is available in the articles themselves. Since some journals have fewer than five articles in all, this resulted in a sample of 205 articles obtained from 47 journals. For the calculations of speed of publication we produced both means and medians, since we noticed a few outlier articles with very long delays.

### Data analysis

The analysis in this study focused on descriptive statistics using Excel. The collected sample data were used to estimate the total number of active journals and the total predatory OA publication volumes between 2010 and 2014 across different strata and overall as well as the annual average number of articles published per journal.

In view of the use of stratified multistage sampling, the following formula was applied to calculate the population total

$$ {\widehat{\gamma}}_{st}={\varSigma}_{i=1}^L{\varSigma}_{j=1}^{n_i}{W}_{ij*{y}_{ij}} $$

and mean

$$ \widehat{\mu}=\frac{{\widehat{\gamma}}_{st}}{{\displaystyle {\sum}_{i=1}^L}{\displaystyle {\sum}_{j=1}^{n_i}}{W}_{ij}} $$

where ${\widehat{\gamma}}_{\mathrm{st}}$ is the estimated population total, *L* is the number of strata, *n*_*i*_ is the total sample size of stratum *i, W*_*ij*_ is the sample weight for the *j*^*th*^ observation in the stratum *i*, *y*_*ij*_ is the value of unit *j* in stratum *i* and $\widehat{\mu}$ is the estimated population mean.

Since journals for all 20 publishers in the 100+ strata were sampled and the total number of journals per publisher were known, we calculated the total article volume for the stratum by multiplying the average number of articles per journal for each publisher with that publisher’s number of journals, and then summing up the results over the 20 publishers.

Regarding the statistical reliability of our results, we calculated the standard error for key mean estimates for the 95 % confidence level. Since we did not use fully random samples, it was not possible to obtain the exact standard error of means; however, what we could do was to provide approximate standard errors for a few results. The standard error is defined as an estimate of the standard deviation of a sampling distribution. We could compute standard error (SE) under the total population size *N*, the population size *N*_i_ in stratum *i*, the sample size *n*_*i*_ in stratum *i*, the sample estimate of the population standard deviation *s*_*i*_ in stratum *i* by the following formula [21]:

$$ SE=\left(\frac{1}{N}\right)*\sqrt{{\displaystyle \sum_{i=1}^L}\left[{N}_i^2*\left(1-\frac{n_i}{N_i}\right)*\frac{s_i^2}{n_i}\right]} $$

In order to identify whether the average APC and publishing speed of the four publisher strata were actually different from each other, we calculated the *P* value under a statistically significant t-test at the 5 % significance level. If the attained *P* value was larger than 5 % then there was no significant difference between the two groups, and vice versa.

### Limitations

Due to the complexity of our sampling method, our results should be treated only as rough estimates showing the overall magnitude of predatory publishing and its central aspects. However, we still believe that our choice of method does not significantly affect the interpretation of the results. The diversified results we obtained for different strata seem to warrant our choice.

## Results

In the reporting below we provide both the results within each stratum and the results generalized to the whole population, where the stratum sizes in terms of journals have been taken into account. Particularly for estimating the average number of articles per journal and APC level, we excluded empty journal websites from the calculations.

### Number of journals

We found 11,873 journals, published by 996 publishers (of which 447 publish just one journal). Of these journals, we estimate that around 67 % (around 8,000 journals) were active, in the sense that they published at least one article. The share of empty journal websites was particularly noticeable among the journals from publishers with journal portfolios of 100 or more journals (46 %) and much lower in the smaller publisher strata (10–99, 23 %; 2–9, 18 %; and single, 2 %). The problem of empty placeholder journals is a problem specific to predatory journals.

**Fig. 2:**
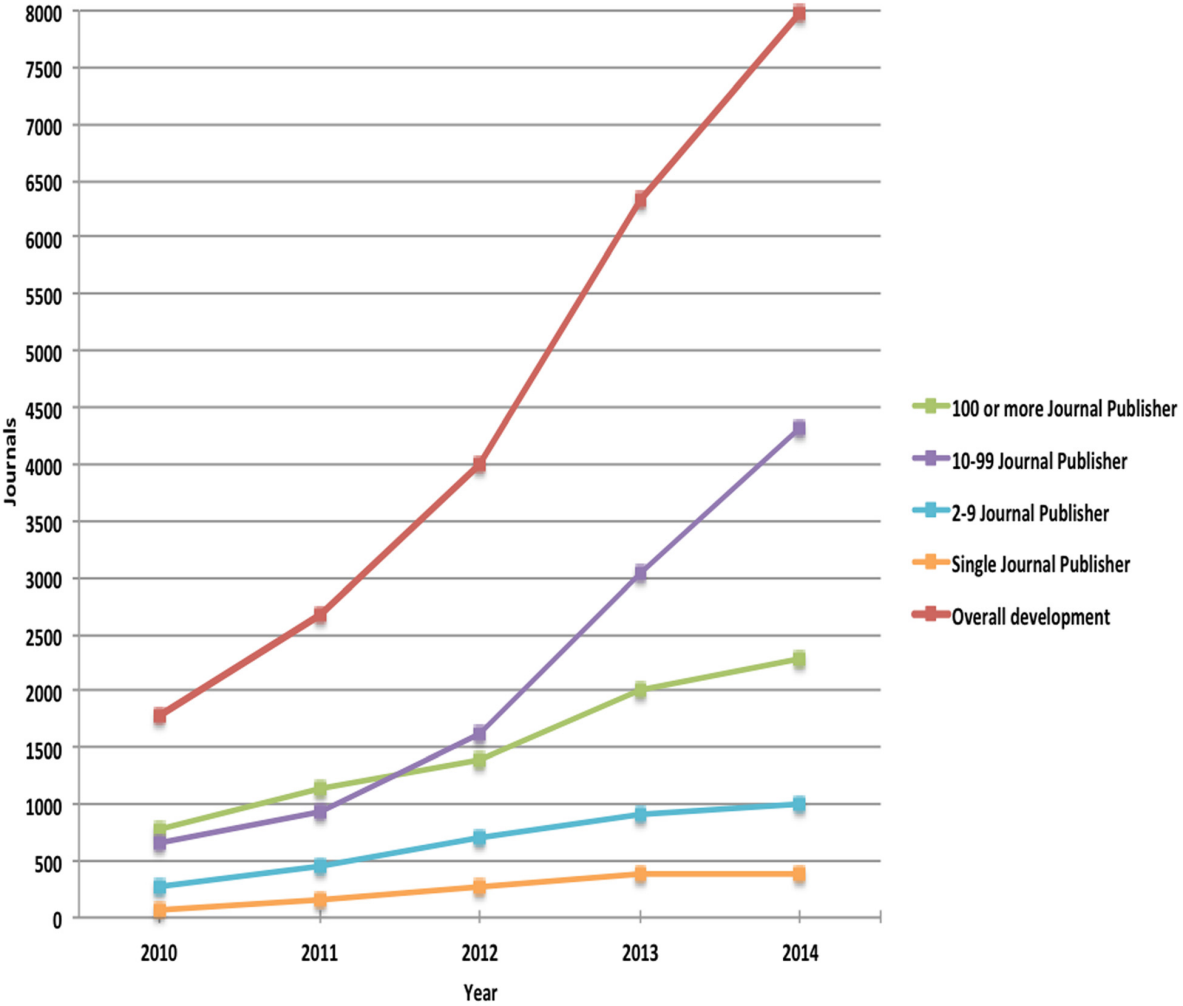
The development of active predatory open access journals from 2010 to 2014

Figure 2 provides the overall development of journal volumes over time and for the different strata. The total number of active journals has grown rapidly from an estimated 1,800 journals in 2010 to around 8,000 journals in 2014. Growth has been particularly strong in the 10–99 stratum.

### Article volumes

**Fig. 3:**
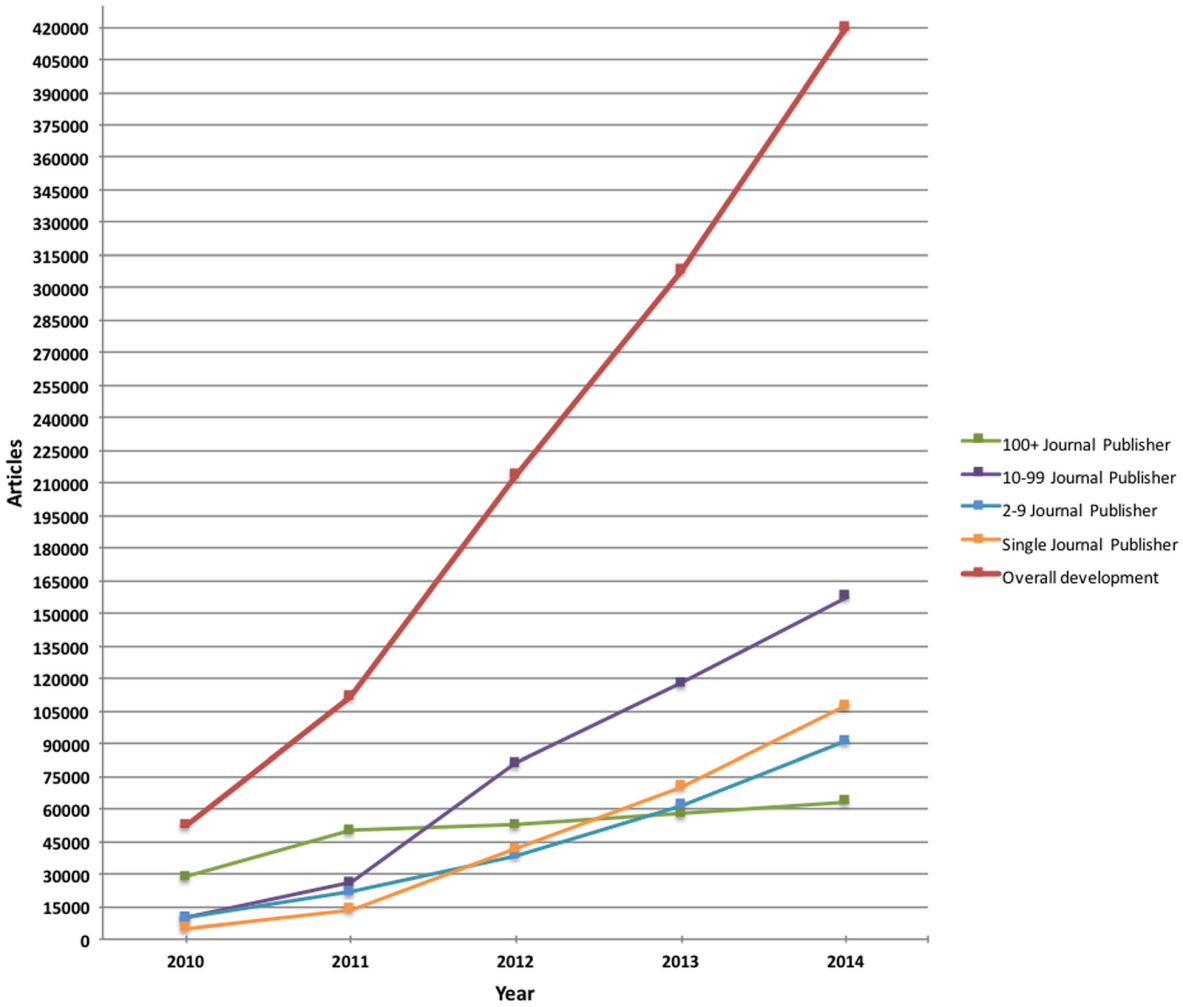
The development of predatory open access article volumes from 2010 to 2014

In total these journals published an estimated 420,000 articles in 2014, after a relatively linear growth from 53,000 in 2010 (Fig. 3). The large publishers dominated the market in 2010 and still in 2011, but after that their absolute article numbers only increased slightly. In 2012, journals from the 10–99 stratum rapidly took over market domination and have consolidated that position even when the two smallest strata also showed continuous fast growth.

### Distribution over scientific disciplines

**Fig. 4:**
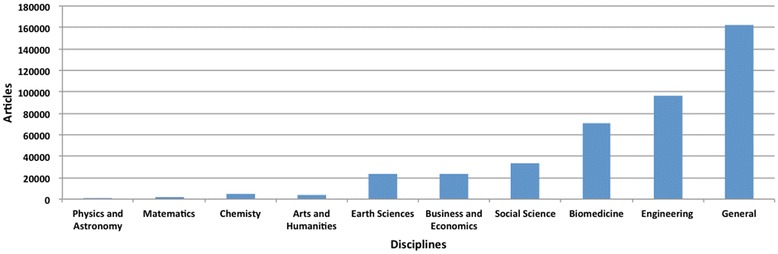
The distribution of predatory open access articles in 2014 by scientific discipline

Figure 4 presents article volumes published in 2014 by journals from different scientific disciplines. The article volumes in journals categorized as ‘general’ were largest with an estimated 162,000 articles. A more detailed analysis would require classifying articles in ‘general’ journals into some of the other subcategories, which was beyond the resource limitations of this study. Quite noticeable from the figure is the large share of articles in engineering journals (97,000 articles), followed by biomedicine with around 70,000 articles.

### Average number of articles per journal

**Fig. 5:**
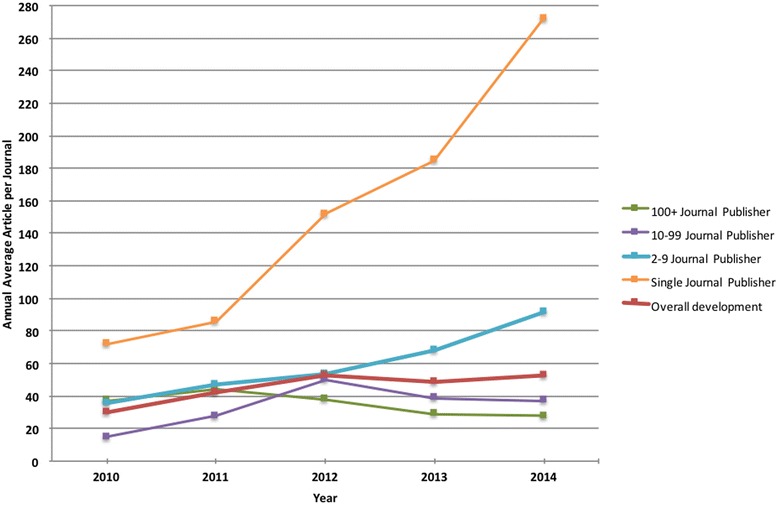
The development of the average number of articles per journal and year from 2010 to 2014

Figure 5 shows the development of the average number of articles per year. The overall averages grew from 30 articles per journal in 2010 to 53 articles in 2012, but after that the number seems to have stabilized. The overall average in each stratum conceals the fact that the average is much higher for single-journal and 2–9 publishers than for the two uppermost strata.

### Country of publishers

**Fig. 6:**
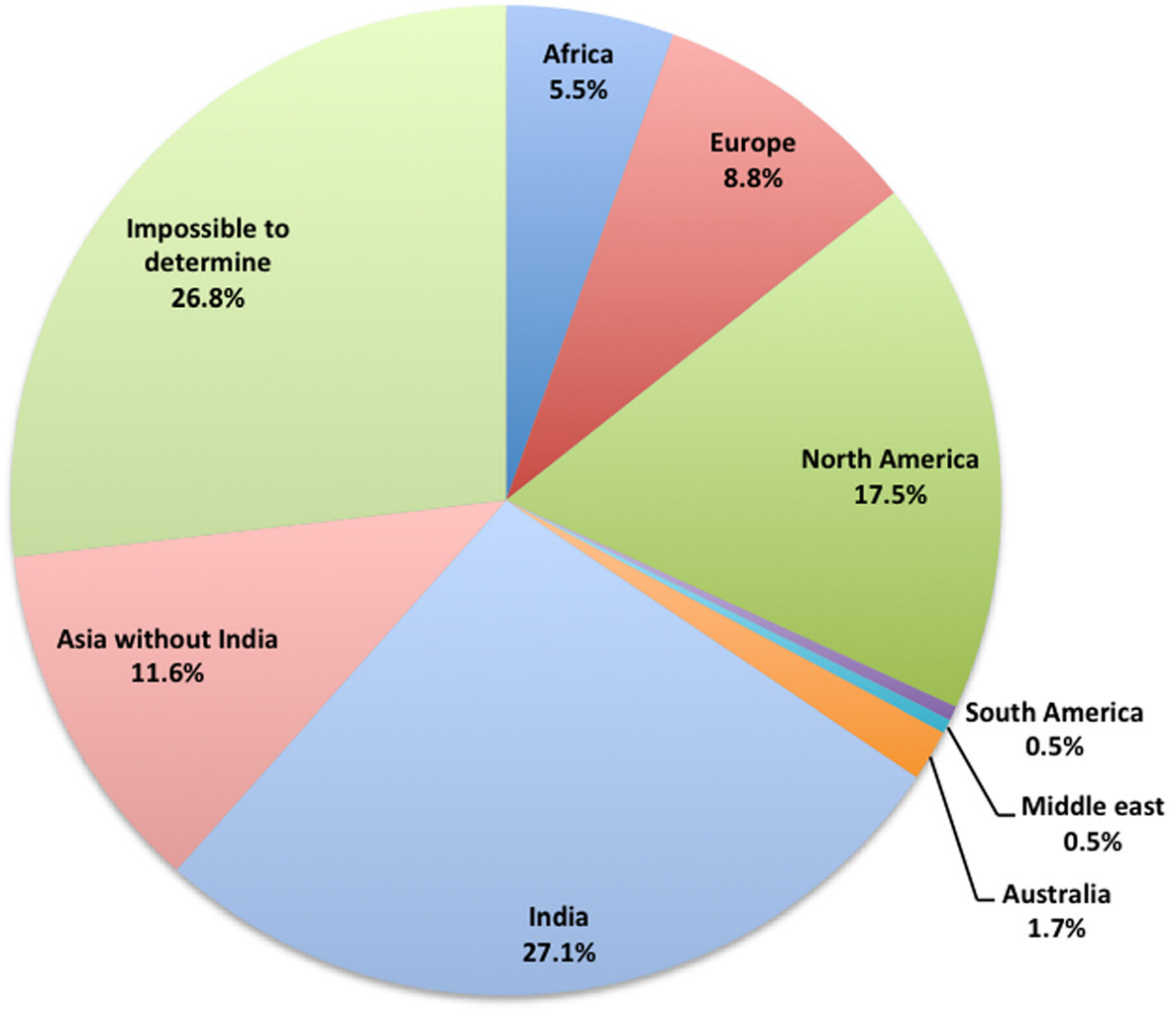
The distribution of publishers (n = 656) by geographic regions

Figure 6 describes the distribution of the publishers across geographic regions. The distribution is highly skewed, with 27 % publishing in India. A total of 52 publishers quote addresses in several countries, for instance, often a combination of the USA or a Western European country with a country from Africa or Asia. In order to establish how credible a USA/European address was, we took a closer look at the 3D street view of the address using Google Maps. If the result was a location that was not credible or, for instance, a PO Box, we classified the journal according to the alternative address. For some addresses that were very difficult to identify, we put them in the category of ‘impossible to determine’.

**Fig. 7:**
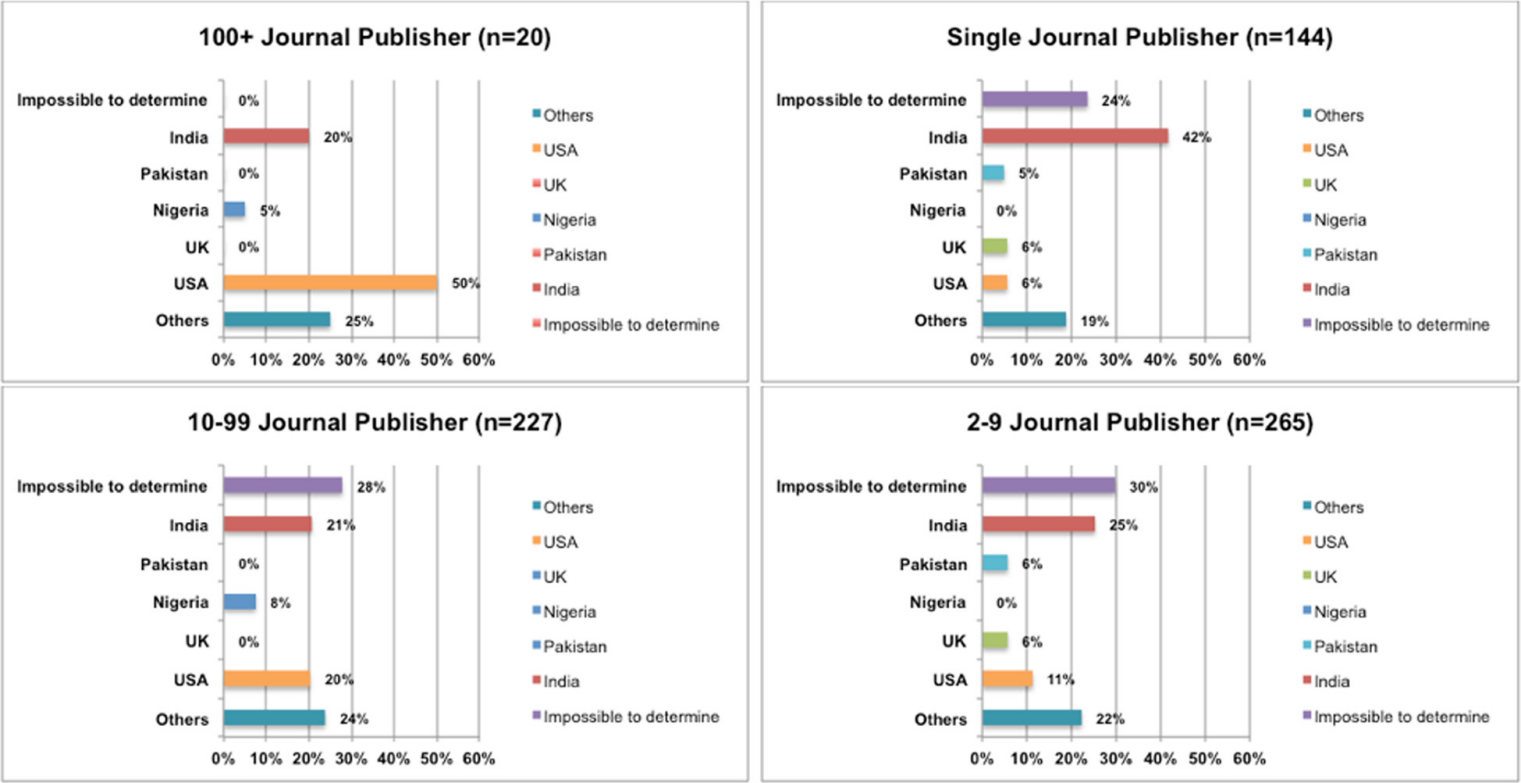
The distribution of publishers by country for the different strata

Figure 7 provides information about how publishers are distributed in each stratum. India dominates the single-journal publisher stratum where the share is 42 %.

### Country of authors

**Fig. 8:**
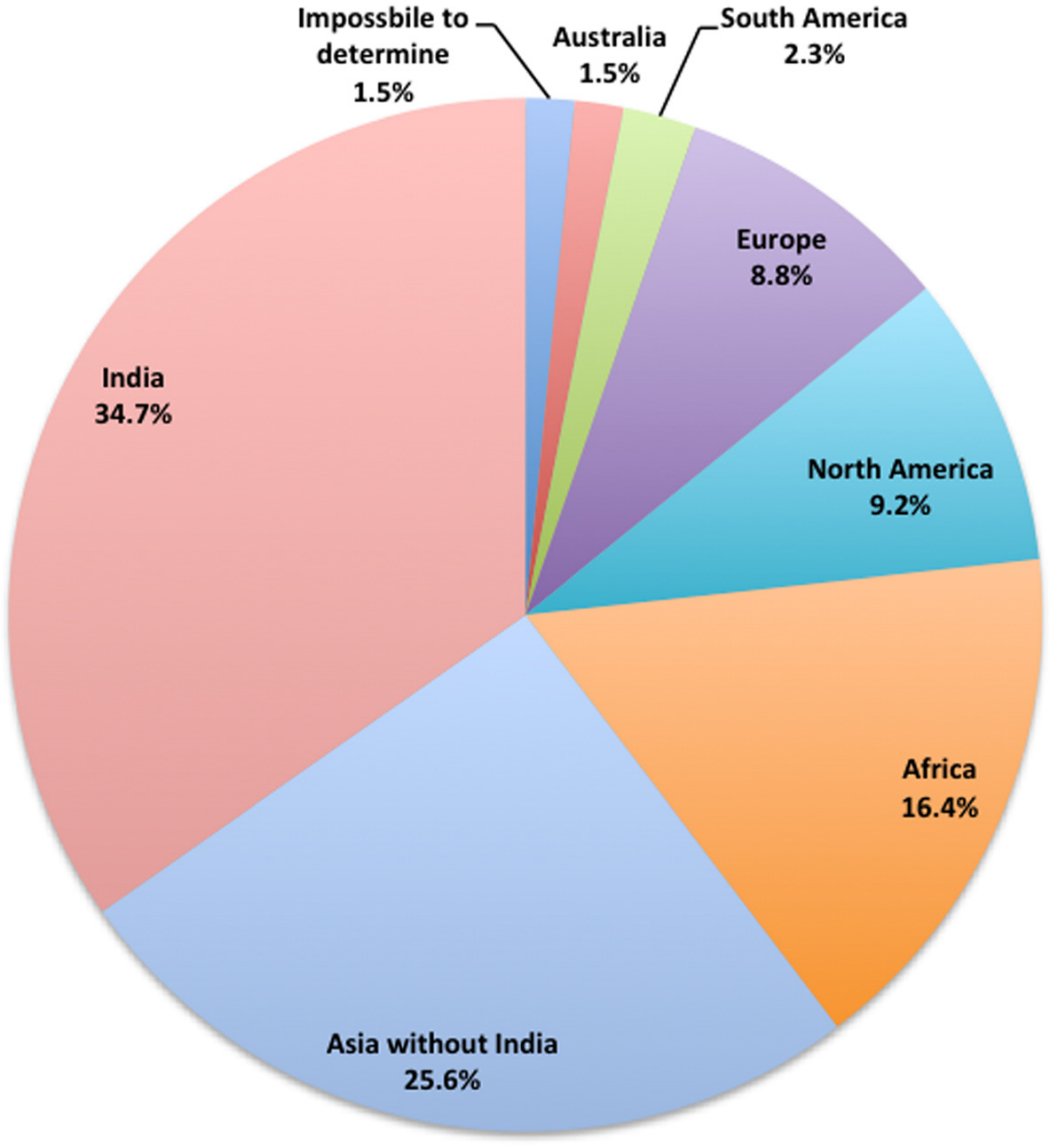
The distribution of the corresponding authors by geographic regions

Figure 8 describes the regional distribution of the 262 sampled corresponding authors, which is highly skewed to Asia and Africa. Around 35 % of authors are from India, followed by Nigerian authors (8 %) and US authors (6 %).

### APC levels

There are clear differences in the APCs of the large and small publishers (*P* <0.05), as is shown in Table 1, with the large publishers operating more expensive journals.

**Table 1:** Average APC for journals and articles published from 2010 to 2014

Publisher stratum	Average APC for journals in USD	Average APC for articles published (2010–2014) in USD
100+ journals	605	796
10–99 journals	239	104
2–9 journals	215	133
Single-journal	98	83
Total	304	178

**Fig. 9:**
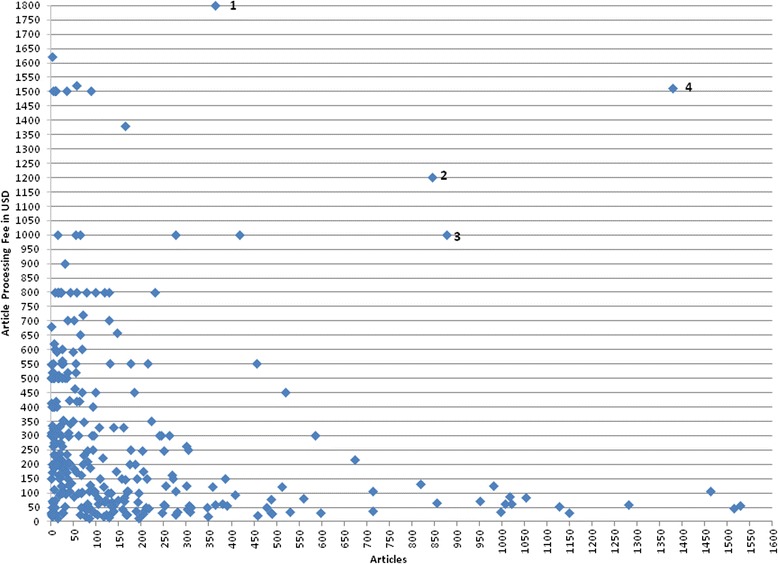
Scatter plot of article numbers versus article processing fee

We calculated the results in two ways. Firstly, by just a direct average (each journal having equal weight). Secondly, by assigning each journal a weight according to the number of articles published in the past five years. The latter calculation better reflects the average APCs paid by authors publishing in these journals. The results turn out quite different depending on the calculation method, in particular for the 10–99 stratum, where the average declines from 239 USD per journal to only 104 USD per article. Also generalized to all predatory articles, the overall average APC is only about half as high (178 USD) per article as the average calculated over journals, indicating a clear author preference for lower priced journals, leading to higher publication volumes. The distribution of APCs as a function of the article volumes in the scattergram (Fig. 9) also illustrates this pattern.

In the scatterplot of the sampled journals, there are four outlier journals (indicated by numbers), which break the clear pattern of diminishing article volumes as a function of increasing APCs. Journal 1, 3 and 4 are published by large publishers (100+), and in particular journal 4 (*Remote Sensing*), which sticks out the most, has a JCR impact factor of 2.6. Journal 2 is the ‘hijacked’ journal *Experimental & Clinical Cardiology*, which in 2014 still retained its impact factor.

### Publishing speed

The average and median publication time for journals and articles published in 2014 (the two measures were calculated in the same way as for APCs) were calculated. The results show that predatory publishers take an average of 3.6 months to publish if we calculated over journals and 3.3 months weighted by number of articles in 2014. However, we consider the median (2.7 overall) a more meaningful metric, since that eliminates the effects of a few outlier articles with very long delays.

### Standard errors and t-tests of the results

The standard errors for some of the key results are presented in Table 2. Likewise the t-tests for some of the stratified data are included in Table 3. Based on the t-tests, we did not find a significant difference among the four publisher strata in terms of their publishing speed (*P* >0.05), so it was not meaningful to report the stratified results but we reported only the total numbers.

**Table 2:** The standard error for article volumes in 2014, average number of articles per journal, average APC for journals and articles, and average and median publication time for journals and articles

Statistics summary	Estimated value		Standard error (a = 95 %)
Total article volumes published in 2014	419,273		90,954
Average number of articles per journal	2010	30	5
	2011	42	9
	2012	53	13
	2013	49	9
	2014	53	8
Average APC for journals in USD	100+ journal publisher	605	41
	10–99 journal publisher	239	26
	2–9 journal publisher	215	24
	Single-journal publisher	98	16
	Overall	304	20
Average APC for articles published (2010–2014) in USD	100+ journal publisher	796	44
	10–99 journal publisher	104	15
	2–9 journal publisher	133	14
	Single-journal publisher	83	17
	Overall	178	17
Average publication time for journals in months	100+ journal publisher	4.4	0.9
	10–99 journal publisher	2.2	0.4
	2–9 journal publisher	3.4	0.8
	Single-journal publisher	3.9	0.7
	Overall	3.6	0.4
Average publication time for articles published (2014) in months	100+ journal publisher	2.9	0.3
	10–99 journal publisher	2.2	0.3
	2–9 journal publisher	4.2	0.7
	Single-journal publisher	4.6	0.9
	Overall	3.3	0.2
			Interquartile range
Median publication time for journals in months	100+ journal publisher	2.6	(2.1, 4.5)
	10–99 journal publisher	2.1	(1.0, 3.0)
	2–9 journal publisher	3.7	(1.8, 4.7)
	Single-journal publisher	3.2	(2.6, 5.2)
	Overall	2.7	(2.0, 4.2)
Median publication time for articles published (2014) in months	100+ journal publisher	2.4	(1.5, 4.3)
	10–99 journal publisher	1.9	(1.1, 3.3)
	2–9 journal publisher	4.2	(2.6, 5.2)
	Single-journal publisher	3.4	(1.8, 5.1)
	Overall	2.7	(1.5, 4.5)

**Table 3:** T-tests for average APC and publication time for journals and articles

	100+ journal publisher	10–99 journal publisher	2–9 journal publisher	Single-journal publisher
Average APC for journals				
100+ journal publisher	-	*P* <0.05	*P* <0.05	*P* <0.05
10–99 journal publisher	-	-	*P* >0.05	*P* <0.05
2–9 journal publisher	-	-	-	*P* <0.05
Single-journal publisher	-	-	-	-
Average APC for articles published in 2010–2014				
100+ journal publisher	-	*P* <0.05	*P* <0.05	*P* <0.05
10–99 journal publisher	-	-	*P* >0.05	*P* >0.05
2–9 journal publisher	-	-	-	*P* <0.05
Single-journal publisher	-	-	-	-
Average publication time for journals				
100+ journal publisher	-	*P* >0.05	*P* >0.05	*P* >0.05
10–99 journal publisher	-	-	*P* >0.05	*P* <0.05
2–9 journal publisher	-	-	-	*P* >0.05
Single-journal publisher	-	-	-	-
Average publication time for articles published in 2014				
100+ journal publisher	-	*P* >0.05	*P* >0.05	*P* <0.05
10–99 journal publisher	-	-	*P* <0.05	*P* <0.05
2–9 journal publisher	-	-	-	*P* >0.05
Single-journal publisher	-	-	-	-

## Discussion

Our use of Beall’s list of predatory publishers as the main external data source can be questioned, since the list is highly controversial. Our choice of using it as a starting point for data collection was dictated by practical resource constraints. Nevertheless, the process of searching the websites demonstrated tangibly to us that the publishers and sampled journals usually fulfilled several of Beall’s criteria, although we did not systematically record our impressions. The multi-tier sampling method used was the most realistic option to keep the time used for manually searching for data reasonable, and also to enable us to study the variations between different publisher strata, which proved to be considerable.

Our estimate of the number of predatory journals is comparable to the 10,606 journals currently (7 June 2015) included in the DOAJ. The overlap is relatively minor. We estimate that 7.8 % of journals from Beall’s list are indexed in the DOAJ (the index recently tightened its inclusion criteria). The overall volume of articles published in predatory journals is also of the same magnitude as in the journals indexed in DOAJ. Laakso and Björk [22] estimated that number to be 340,000 in 2011, and extrapolating the growth would have meant roughly half a million in 2014. For comparison, the number of articles published in ISI-indexed journals was estimated to be 1,033,000 in 2009 [23].

Until 2012, the growth in predatory article numbers occurred mainly through publishers who set up large (100+) journal portfolios, and who on average charge almost 800 USD, but during the past three years the 10–99 journal publishers, who on average charge only 104 USD, have started to dominate the market.

The average number of articles per year in predatory journals (around 50) is comparable to publishing volumes in DOAJ-indexed OA journals, where the average yearly number of articles has slowly risen and was 40 articles per journal in 2009 [23]. Due to the emergence of megajournals, the average is likely to be higher today. Björk et al. estimated the average number in ISI-indexed journals (mostly subscription) to be 111 in 2007 [25].

Growth in article numbers within predatory journals has in the past two years mainly occurred in the two lowest strata, which tend to have much higher annual publication volumes. Indian journals have a strong position especially in the single-journal stratum.

Our data showed a big difference in APC levels depending on the stratum. The APCs by predators are, nevertheless, much lower than the APCs by more credible OA publishers, which on the other hand often offer waivers from the charges to authors from developing countries. The average of DOAJ journals with APCs is around 900–1,000 USD [26, 27]. Currently leading universities in the UK and Germany, which fund APCs centrally, tend to pay on average 1,200–1,300 USD [28].

Using our data for the number of articles and average APC for 2014, our estimate for the size of the market is 74 million USD. The corresponding figure for OA journals from reputable journals has been estimated at 244 million USD in 2013 [29]. The global subscription market for scholarly journals is estimated to be around 10.5 billion USD [30].

A study by Solomon and Björk [31] about the sources of funding for the APCs showed that in the case of authors from countries with a GDP per capita of over 25,000 USD, only 10 % of the APCs came from personal funds, whereas the proportion for authors from developing countries (under 25,000) was 39 %. That study concerned DOAJ-indexed OA journals of relatively good reputation, a third of which with JCR impact factors. If authors from low-income countries to a large extent need to pay the APC out of their own pockets, then this explains the generally low average of 178 USD and the fact that predatory journals with lower prices tend to have grown much faster recently.

Our results concerning the regional distribution of authorship can be compared with the results of Xia et al. [18] who studied the authorship distribution for seven pharmaceutical predatory journals, and Ezinwa Nwagwu and Ojemeni [19] who studied 34 journals from two Nigerian-based predatory publishers. The minor differences in the results can be explained by the much more limited journal samples in the above studies, for instance, the journals studied by Ezinwa Nwagwu and Ojemeni [19] had an average APC of 636 USD, which could explain the lower share of Indian authors.

An interesting finding is the very low share of South America, both among publishers (0.5 %) and corresponding authors (2.2 %). It would no doubt be an interesting question to study the reasons for this, which could be a combination of factors, where the infrastructure in Latin America differs from countries like India and Nigeria.

Above we have reported the estimated geographical spread of predatory article authorship in terms of absolute numbers per year of articles, which is highly skewed with India at the top. A slightly different viewpoint would be a per capita calculation, which takes into account the relative sizes of countries or economies. In our view a particularly interesting comparison is one in which the size of predatory publication is compared to the production of high quality article from the same country. We used figures from the Web of Science (InCites regions report) about authorship for the years 2013–2014 to calculate the ratio of predatory to Web of Science-indexed articles. For the four biggest contributors of predatory articles, the USA had a low ratio of 6 %, Iran 70 %, India 277 % and Nigeria a staggering 1,580 %.

The publishing delays we found were much shorter than for scholarly journals in general. Björk and Solomon [32] found delays of 9–18 months, depending on the field of science, with social sciences having the longest delays. The average delay for the OA journals in that study was 5.9 months, thus clearly shorter than for subscription journals but longer than for predatory journals. The range of average delays for OA megajournals was 3–5 months.

Unlike many writings about the phenomenon, we believe that most authors are not necessarily tricked into publishing in predatory journals; they probably submit to them well aware of the circumstances and take a calculated risk that experts who evaluate their publication lists will not bother to check the journal credentials in detail. Hence we do not uncritically see the authors as unknowing victims. The universities or funding agencies in a number of countries that strongly emphasize publishing in ‘international’ journals for evaluating researchers, but without monitoring the quality of the journals in question [16, 33], are partly responsible for the rise of this type of publishing. The phenomenon should probably, however, be seen more broadly as a global North-South dilemma where institutions in developing countries are unable to break free from the increasingly globalized and homogenized view of academic excellence based on ‘where’ and how often one publishes, instead of ‘what’ is published and whether the results are relevant to local needs. In that sense, these authors and their institutions are part of a structurally unjust global system that excludes them from publishing in ‘high quality’ journals on the one hand and confines them to publish in dubious journals on the other.

Leading respectable OA publishers have not stood by silently as OA has been given a bad name by predators. Rather than blacklisting journals, which Jeffrey Beall is doing, the strategy has been one of defining quality criteria and accreditations of journals that meet those [34]. For instance, the DOAJ has, since 2014, imposed stricter criteria for inclusion and has filtered out journals that do not meet them [35]. Membership in the Open Access Scholarly Publishers Association (OASPA) is also contingent on meeting quality criteria. An increasing share of respectable OA journals is also nowadays indexed by the ISI.

We are not particularly satisfied with the term ‘predatory’, since we believe that the term has a highly negative connotation and we feel it is slightly misleading. We would instead have preferred to talk of ‘open access journals with questionable marketing and peer review practices’. Nevertheless the term ‘predatory’ open access is by now so established for this phenomenon that in the end we decided to use it. A practical consideration is that an article using the term in the title or frequently in the text is more likely to be picked by readers searching the internet for more information about this phenomenon.

## Conclusion

In this study, we used a multistage stratified sampling method to take a look into the predatory publishers and journals on Beall’s list and generated their development trend over time. We found that the problems caused by predatory journals are rather limited and regional, and believe that the publishing volumes in such journals will cease growing in the near future. Open access publishing is rapidly gaining momentum, in particular through the actions of major research funders and policy makers. This should create better opportunities for researchers from countries where predatory publishing is currently popular, to get published in journals of higher quality, in particular since most journals have a policy to waive the APCs for authors from developing countries.

## Abbreviations

APC: Article processing charge
DOAJ: Directory of Open Access Journals
ISI: Institute for Scientific Information
ISSN: International Standard Serial Number
JCR: Journal Citation Reports
OA: Open access
OASPA: Open Access Scholarly Publishers Association
SE: Standard error
